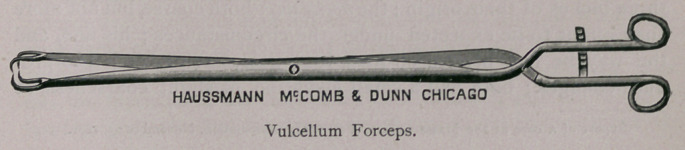# Sterility of Mares

**Published:** 1892-01

**Authors:** M. E. Knowles

**Affiliations:** State Veterinarian of Indiana; Terre Haute, Ind.


### STERILITY OF MARES.

By M. E. Knowles, D. V. S., State Veterinarian of Indiana.

Sterility of the mare and cow has attracted the attention of breeders in particular, and veterinarians in a mild sort of way, for years. There have been many instruments invented and proposed for the cure of sterility, without a single suggestion as to the probable cause, other than ridged Cervix—"Contracted Os." I will not offer suggestions as to the merits of the different impregnators and nostrums offered to the breeder for the POSITIVE cure of sterility, but leave this for your individual opinion.

To my knowledge, no veterinarian has described any of the most frequent causes of temporary sterility, and it is with much hesitation that I now attempt it. I assume that all are acquainted with the anatomical relations and physiological process of procreation, and go directly to one of the most frequent causes of temporary sterility. There is a popular idea that the semen is ejaculated directly through the cervical canal into the uterus, and without this conception cannot occur. This is a physiological impossibility, and, if it ever occurs, is purely by accident and due to an extremely flacid cervix with a uterus ballooned by air.

To disprove this popular theory, during the breeding season of 1887, through the kindness of Mr. M——, I conducted a series of experiments on seven brood mares in the following manner: A few moments before copulation, in each mare, the vaginal speculum was introduced, and the cervix secured with a vulcellum forceps; a rubber band was then tightly drawn about the body of the cervix; a stallion serving the mares in from two to fifteen minutes after the application of rubber band; ten minutes later the band was removed by introduction of the hand, and in five of the seven mares conception followed the first service; one was served twice and one three times before conception occurred, but at each service the elastic band was tightly applied. Perhaps a more interesting and convincing proof of the absurdity of the intera-uterine-semeninjection hypothesis may be seen in the following: In June of 1888, a mare, aged four years, thirteen and a half hands, was presented to a large, standard bred stallion for service. The horse, after two ineffectual attempts to complete the sexual act, refused to again mount her, although the penis entered about half its length each time. I was called a few hours later to see her on account of apparent colic, with which she had suffered since the attempted copulation. On introducing the vaginal speculum, the cervix was found intensely hyperæmic, evidently from contusion with the glans penis. On measurement, the vagina was found to be only eleven inches in length. This shortness of the vagina readily accounted for the inability of the horse to complete sexual congress and the cervical hyperæmia. This mare being of some value on account of "blood lines," the owner was anxious to have her in foal, and to accomplish that end I suggested that the horse be permitted to serve another mare during the following œstrum of his mare, and that I be allowed to transfer the semen by mechanical means to the mare in question. During the following month this was accomplished; the mare conceived and completed a successful gestation. In this instance the semen was transferred with a curette, the temperature being maintained through the medium of hot water.

From the above-mentioned and other experiments, I am led to the following conclusions: First, in a perfect physiological condition of the mare, the zoasperms reach the cavity of the uterus by their own movements; second, the least pathological condition may, and often does, obstruct the movement of the zoasperms, thereby preventing their entrance into the uterus; and farther, that certain secretions, the products of pathogenic conditions, destroy the vitality of the zoasperms, thereby preventing their passage through the vagina and cervical canal, and should they reach the uterus, they then encounter pathogenic secretions that destroy them.

Such acute and chronic cervical hyperæmiæ are probably the most frequent and fruitful causes of temporary sterility, due in an astonishingly large number of instances to continually recurring abortions. By recurring abortions, I mean that the abortions occur regularly after each conception within from twenty-one to sixty days, and this fact is common to both mares and cows, but on account of the very small fœtus, escapes the notice of the attendant, even if passed on to a clean floor, which is rarely the case.

Suspecting the above facts several years ago (1886), I commenced to obtain information relative thereto, by making careful enquiry of all intelligent breeders of my acquaintance. I found, however, that not one of them even suspected such an event.

Mr. McK——, a very intelligent breeder of cattle, had at this time two cows that were continually being bred at an interval of about two months, which he very kindly offered me for observation. He gave me the date of last service of each, and ten days from these dates I commenced making two examinutions daily by speculum of each cow. On the morning of the 29th day from date of service, in No. 1 I found in her vagina a twenty-nine-day-old fœtus. In cow No. 2, on the morning of the forty-third day, the attendant, by "some peculiar circumstance," found a forty-three-day-old fœtus behind her, which he presented to me on my arrival at the farm. During this and the following year, it was my fortune to make the same observations on twelve brood mares from different farms, with the result of positively finding the infinitesimal fœtus in the vagina of nine of the twelve, from twenty-one to sixty-seven days old.

In the remaining three the foetus escaped me, but the condition of the cervix and intra uterine mucosa pointed plainly to the fact of abortion having occurred.

I will not venture an opinion as to the probable cause of the primary abortion, unless it is due to complete or patchy hyperæmia of the endometrium or cervix ; the conditions most frequently found existing, subsequent to the abortion, and in mares not known to have aborted. The condition of acute cervical hyperæmia will be easily recognized on introducing the vaginal speculum; the cervix, and frequently the adjacent vaginal mucus membrane, will be observed engorged, vessels distended and the cervical folds thicker than in health; glued together by a thick mucus or muco purulent discharge, which is rather scant. The thickening of cervical folds at this time is evidently due to blood infiltration but should the hyperæmia continue for some days the cervical connective tissue becomes infiltrated with serum and the folds oedematous, in which condition it remains for some days longer, when the exudation may become plastic; there sometimes seems to be a local lymphangitis, and occasionally, when this occurs, from the interference in circulation, or possibly blocking of the lymph vessels, there occurs a local circumscribed necrosis of the cervical mucus membrane leaving an ulcer, or ulcers, that are tardy healing.

The subject of vaginal and cervical ulcer will be considered in a later paper, when I shall attempt to consider cervical hyperæmia in a more extended manner. Cervical hyperæmia is usually only a temporary cause of sterility, and is by change of surroundings and climate, or more freqently from keeping the mare away from the stallion during three or four rutting periods relieved, and, on subsequent service the mare conceives; and should there be no pathological condition remaining, accomplishes a successful gestation.

This desired event can, however, be, by the veterinarian's assistance, hastened in many instances. The treatment must be governed by indications alone; hot or cold vaginal irrigations, with the addition of from five to fifteen per cent. boracic acid; tamponing cervix with bolted boracic acid, etc. When there is intense hyperæmia, I have found tamponing the cervix with a large pad of absorbent cotton, shaped to fit the cul-de-sac and completely enveloping the cervix, to be of material benefit, promptly relieving the hyperæmia by depletion. This tampon should have a string attached by which the attendant may remove it in from five to eight hours after insertion. Where it is necessary to adopt this method, I follow the tampon for several days with a warm boracic acid water, vaginal injection, employing usually from two to four gallons of water, at a temperature of 100 F.

In chronic hyperæmia, accompanied by cellular changes, and an organized exudate; painting the cervix once or twice weekly with Comp. Tr. Iodine, followed with daily hot vaginal irrigations, has, in my hands, been productive of much good.

There is much in an examination properly made, and perhaps it may not be untimely for me to call your attention to the accompanying cuts of some gynæcological instruments, necessary for all veterinarians in breeding districts, who are desirious of seeing "per vaginam" what there is in this direction. The instruments shown are necessary for the successful practice of gynæology. These instruments are very nicely and lightly made by Haussmann & McComb, of Chicago, who have, by my direction, succeeded in getting all, but most especially the vaginal speculum, so light and convenent that its introduction and retention is born without inconvenience by the most nervous mare. The cervical speculum is used for intra uterine examinations, and should be introduced before the vaginal.

The method of introducing the cervical speculum is as follows: The hand and arm well oiled is introduced, and the cervix dilated, then thoroughly oil the speculum, introduce and, by depressing the handles, open the valves to a convenient width; now introduce the vaginal speculum, having the mare in a convenient sun-light, and the wide, brightly-polished single valve will reflect ample light into

### KNOWLES' VETERINARY GYNAECOLOGICAL INSTRUMENTS.

Cervical Speculum.

Vaginal Speculum.

Applicator.

Curette

Applicator Forceps.

Vulcellum Forceps.

the uterus for ordinary examination; should, however, a more minute examination be required, resort must be had to a small electric light of three or four candle-power, power furnished by a portable storage battery, which can be carried into the uterus with the applicator or applicator forceps. For the purpose of facilitating the reflections of sun-light into the uterus, it will be necessary to manipulate the cervical speculum with the end of the vaginal speculum to the desired angle; this can, however, be more satisfactorily accomplished by securing the cervical speculum in the vulcellum forceps, thereby allowing perfect liberty of the vaginal speculum, and consequently better light reflection.

As time may permit, I will present you with some other causes of sterility, some of them very rare and interesting, and report some cases treated during the last year.

TERRE HAUTE, IND., Dec. 12, 1891.

## Figures and Tables

**Figure f1:**
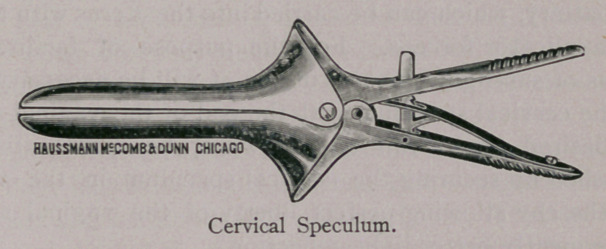


**Figure f2:**
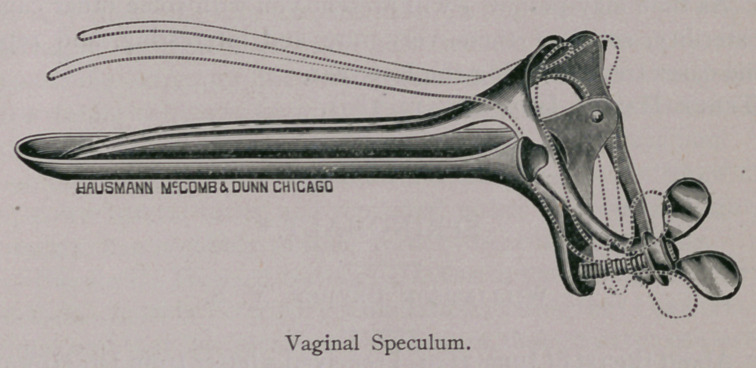


**Figure f3:**
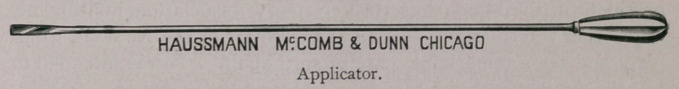


**Figure f4:**
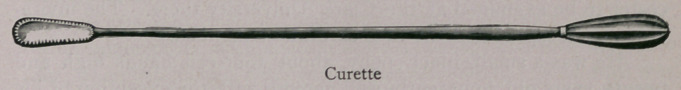


**Figure f5:**
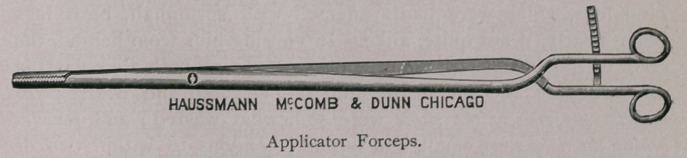


**Figure f6:**